# Does Decreased Vitamin D Level Trigger Bipolar Manic Attacks?

**DOI:** 10.3390/bs13090779

**Published:** 2023-09-18

**Authors:** Okan İmre, Mustafa Karaağaç, Cuneyt Caglayan

**Affiliations:** 1 Department of Psychiatry, Faculty of Medicine, Karamanoglu Mehmetbey University, Karaman 70200, Turkey; 2 Department of Psychiatry, Karaman Training and Research Hospital, Karaman 70200, Turkey; 3 Department of Medical Biochemistry, Faculty of Medicine, Bilecik Seyh Edebali University, Bilecik 11200, Turkey

**Keywords:** bipolar disorder, mania, Vitamin D, calcium, phosphorus

## Abstract

Background: Bipolar disorder is a chronic psychiatric disorder with depression and manic episodes. It is one of the leading causes of disease-related disability worldwide. Despite the presence of various alternative drug options for bipolar disorder, some patients do not adequately benefit from the treatment. Therefore, possible underlying mechanisms need to be clarified. Recently, studies on the relationship between bipolar disorder and vitamin D (Vit D) have attracted attention. Although many studies have found an association between depression and Vit D deficiency, little is known about the relationship between manic episodes and Vit D. The aim of this study was to compare Vit D and related metabolites of bipolar manic episodes prior to treatment, bipolar remission after treatment, and healthy control groups. Methods: This case–control study consisted of 34 bipolar manic episode patients and 34 healthy controls. Disease activity was evaluated with the Hamilton Depression Rating Scale (HAM-D) and Young Mania Rating Scale (YMRS). Firstly, serum 25-hydroxy vitamin D (25-OHD), calcium (Ca) and phosphorus (P) levels of patients in the bipolar manic episode were measured and compared with healthy control. Secondly, serum 25-OHD, Ca and P levels in the euthymic periods of the same patients were measured and compared with healthy control. Results: Bipolar manic episode Vit D levels were lower when compared to healthy controls; while there was no difference in terms of Ca and P levels. There was no significant difference between the bipolar euthymic period patients and the healthy control group in terms of 25-OHD, Ca and P levels. Conclusion: Our results demonstrated low serum Vit D concentrations in the acute manic episode of bipolar disorder. Decreased Vit D level may play a role in the onset of the manic episode, or malnutrition and insufficient sunlight during the manic episode may have caused Vit D deficiency. Future studies are needed to exclude potential confounding factors and to compare all mood episodes.

## 1. Introduction

Bipolar disorder is a chronic disease with manic and depressive episodes and intermittent remissions. Studies have shown that the lifetime prevalence of bipolar disorder can reach up to 5% [1]. It is one of the leading causes of disease-related disability worldwide and among the top ten diseases in terms of economic burden [2]. Deterioration of psychosocial functionality, disability risk and loss of workforce are quite high in bipolar disorder [3]. The life span is shorter than expected [4]. Despite the widespread use of mood stabilizers and antipsychotics for treatment, a group of patients do not respond to these treatments [5]. This situation shows that the pathophysiology of bipolar disorder may have different mechanisms. Studies have consistently focused on the possible role of inflammation [6]. It has been reported that stress may play a role in the pathophysiology of bipolar disorder by causing sleep disorders and triggering neuroinflammation [7]. Although several biochemical parameters related to inflammation in bipolar disorder have been investigated [8], the relationship between manic episodes and Vit D has not been adequately investigated. Even though a few studies point to the possible role of Vit D deficiency in manic episodes [9], further research is needed for consistent results.

Vitamin D exists as a prohormone in nature in two forms: Vit D2 (ergocalciferol) of mostly plant origin and Vit D3 (cholecalciferol) of animal origin. Ninety percent of Vit D is obtained through conversion of the precursor 7-dehydrocholesterol to cholecalciferol (D3) in the skin under sunlight. The remaining 10% is obtained through nutritional intake. D3 is hydroxylated to 25-OHD by the enzyme 25-hydroxylase. Subsequently, 25-OHD undergoes hydroxylation by the enzyme 1-alpha hydroxylase in the kidney to form 1,25-dihydroxy Vit D (calcitriol), the active form of Vit D. Plasma 25-OHD concentration is accepted as the best indicator reflecting the Vit D level of individuals [10]. Insufficient sunlight, malnutrition, seasonal changes and aging might affect Vit D synthesis. Studies have reported that Vit D deficiency is associated with several global diseases, so it is critical to take the necessary precautions [11].

Vitamin D is recognized as a neuromodulator compound as it maintains the balance between anti-inflammatory and pro-inflammatory mechanisms [12]. It has been reported that 1,25-dihydroxy Vit D3, the active form of Vit D, has an anti-inflammatory effect, and its deficiency can trigger inflammatory processes [13]. Tryptophan hydroxylase and tyrosine hydroxylase enzymes regulate 1,25-dihydroxy Vit D3. These two enzymes act as rate limiters in synthesizing serotonin, dopamine, norepinephrine, and epinephrine. In addition, Vit D acts as an antioxidant through inhibition of inducible nitric oxide synthase (iNOS) [14]. Vit D deficiency may play a role in the etiology of psychiatric disorders, especially bipolar manic episodes, through the abovementioned mechanisms. 

Vitamin D deficiency may lead to comorbid psychiatric problems due to the role of Vit D in calcium (Ca) and phosphorus (P) homeostasis [15]. Few studies have evaluated Vit D, Ca and P levels in patients with bipolar disorder. In one study, it was stated that bone mineral density (BMD) is low in premenopausal women with bipolar disorder, and a high plasma P level may be an indicator of low BMD. There was no significant difference in plasma Ca in the study [16].

In light of the above literature, it is crucial to investigate the relationship between bipolar manic episodes and Vit D levels due to its role in pathophysiology and comorbid conditions. Although many studies provide evidence regarding the role of Vit D deficiency in psychiatric disorders, little is known about the relationship between Vit D and bipolar disorder manic episodes [17]. 

As a result, the evaluation of Vit D and related metabolites Ca and P levels in bipolar disorder manic episode patients can provide information about comorbid problems as well as etiopathogenesis. The aim of this study was to compare Vit D and related metabolites of bipolar manic episodes prior to treatment, bipolar remission after treatment, and healthy control groups. Our research findings are essential in setting an example for future biochemical studies.

## 2. Material and Methods

### 2.1. Ethical Approval and Participants

Patients between the ages of 18 and 65 admitted to the Psychiatry Clinic of Karamanoglu Mehmetbey University Faculty of Medicine between 2022 and June 2023 with bipolar affective disorder-manic episodes were included in this study. Bipolar disorder and a manic episode diagnosis was made by a specialist psychiatrist according to the Diagnostic and Statistical Manual of Mental Disorders, Fifth Edition (DSM-5). The Young Mania Rating Scale (YMRS) confirmed whether the patients were in a manic episode. Patients with YMRS > 20 were included in the study. Bone disease and endocrinological diseases that may affect Vit D metabolism, developmental disability, phosphocalcemic metabolism abnormality, those who took Vit D supplements for three months before the evaluation, and those with all types of acute and chronic diseases were excluded from the study. Those currently using anti-inflammatory drugs and pregnant women were excluded from the study. Thirty-four of the 45 manic episodes of bipolar disorder were included in the study. Five people with diabetes, three with hypothyroidism, and three with rheumatological diseases were excluded from the study.

The control group consisted of people who donated to the hospital blood bank. Thirty-four age- and sex-matched individuals were included in the control group. Necessary tests were carried out to show that those who wanted to donate blood in our hospital did not have any chronic diseases, infectious diseases or drug use, and their written consent was obtained. Unlike the patient group, any previous psychiatric disorder, including anxiety and depressive disorder, was accepted as an exclusion criterion in the control group. Written and verbal consent for volunteering was obtained from both groups.

This study was carried out according to the revised version (October 2013) of the Declaration of Helsinki. Ethical approval was obtained for the study from Karamanoğlu Mehmetbey University Faculty of Medicine Clinical Research Ethics Committee (Date: 15 December 2022 No: 11-2022/14).

### 2.2. Design and Procedure

In this observational study, we primarily aimed to compare the Vit D, Ca, and P levels of bipolar disorder manic episode patients before treatment with those of the healthy control group. Subsequent to this, the aim was to compare the Vit D, Ca, and P levels of bipolar disorder euthymic period patients with those of the healthy control group.

Sociodemographic data and clinical characteristics of patients hospitalized with the diagnosis of bipolar disorder manic episodes were recorded with semi-structured forms created by the researchers. Vit D, Ca, and P levels were measured 24 h after hospitalization. Vit D supplementation was given to those with low Vit D levels. Patients in remission were discharged. After the same patients were discharged, their Vit D, Ca, and *p* values were measured again after at least 2 months of the euthymic period. All participants gave blood samples and completed the study. There was no patient who could not give a second blood sample and was excluded from the study. Whether or not the patients were in the euthymic stage was evaluated with the YMRS and Hamilton Depression Rating Scale (HAM-D). Those with HAM-D < 7 and YMRS < 4 were considered euthymic. The 17-item HAM-D was used in the assessment of depressive symptoms; The reliability and validity study of the Turkish version was performed by Akdemir, et al. [18]. The YMRS was used to assess manic symptoms; Karadağ, et al. [19] determined the Turkish version’s reliability and validity.

### 2.3. Collection of Blood Samples and Laboratory Analysis

Sterile antecubital venous blood samples were collected in gel-containing tubes and centrifuged for 10 min at 4000 rpm to analyze the separated serum. The 25-OHD was measured using commercially available kits based on routine methods on the Maglumi X3/X8 System (Snipe Diagnostic, Shenzhen, China). Vit D levels between 20–100 ng/mL were considered normal, 10–20 ng/mL was considered to be a Vit D insufficiency, and <10 ng/mL was considered to be a Vit D deficiency [10].

### 2.4. Statistical Analysis

Statistical analyzes were carried out with the R 4.3.1 program. Descriptive statistics of mean and standard deviation for numeric variables, frequency and percentage for categorical variables were given. The Chi-square test was used in the analysis of categorical variables. Independent and paired *t*-tests were used in the analysis of numerical variables. Spearman correlation coefficients were used to examine the relationships between numerical variables. *p* < 0.05 was considered significant.

## 3. Results

When the patient and control groups were compared by age and gender, no statistically significant difference was found (*p* = 0.32, *p* = 0.81) (Table 1). The patient group consisted of 19 women and 15 men. The age range of the patient group was 20–61, while the age range of the control group was 19–58. Vit D levels of bipolar manic episode patients were lower when compared to the healthy control. However, there was no difference between the two groups in terms of Ca and P (respectively; *p* < 0.001, *p* = 0.89, *p* = 0.27) (Table 2). Regarding Vit D, Ca and P levels, there was no statistically significant difference between bipolar euthymic patients and healthy controls. (*p* = 0.58, *p* = 0.44, *p* = 0.86, respectively) (Table 3).

**Figure 1:**
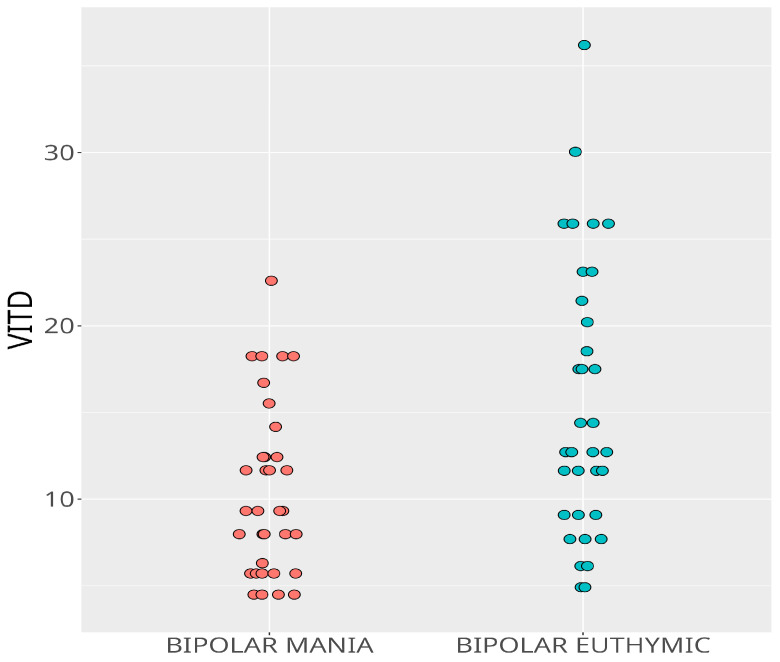
Comparison of serum levels of Vitamin D between bipolar manic episode and euthymic period.

When patients with a bipolar manic episode before treatment were re-evaluated while they were in the euthymic period after treatment, their Vit D levels were found to be increased (Figure 1). There was no statistically significant difference in Ca and *p* values between the manic and euthymic episodes in the patient group (*p* < 0.001, *p* = 0.19, *p* = 0.16, respectively) (Table 4). 

No relationship was found between sociodemographic data and Vit D level. No relationship was found between disease duration, age, number of attacks, or Vit D levels (Table 5). Nineteen (55.8%) of the patients in the manic episode and 10 (29.4%) of the patients in the euthymic period had Vit D deficiency. There was no Ca deficiency or excess Ca in the patient or control groups. Two patients in the patient and control groups had an insignificant P deficiency. (Table 6). 

## 4. Discussion

This study suggests that Vit D deficiency may be associated with manic episodes. In this study, serum Vit D concentration was found to be lower in the acute manic episode of bipolar disorder than in the remission period and the healthy control group. There was no difference between the groups in terms of Ca and P levels. 

Unlike previous studies in the literature, we evaluated serum Ca and P levels, which can be affected by Vit D levels, in the same patients in the euthymic period after treatment. There is no study in the literature comparing the Vit D, Ca, and P levels of the same patients in the pre-treatment manic and post-treatment euthymic periods. One of the main findings of the study, low Vit D levels in the manic episode, is consistent with the results of several studies in the literature. In a study comparing different patients in manic and euthymic periods, Vit D levels were found to be lower in manic episodes compared to healthy controls and euthymic patients [8]. In this study in the literature, the Vit D levels of the manic episode patient group in the post-treatment euthymic period was not evaluated. The euthymic patient group was selected from another bipolar patient group that were outpatients. Therefore, this information in the literature can only provide cross-sectional information. At this point, the longitudinal design of our study may contribute to the literature in terms of providing information about Vit D levels and how other parameters change over time.

In a study comparing the Vit D levels of patients with mood disorders and schizophrenia, it was found that patients with bipolar disorder had lower Vit D levels than patients with schizophrenia [20]. However, Vit D levels in manic episodes were not evaluated separately in this study. No comparison was made between the healthy control group and the post-treatment euthymic status. In this study of the literature, no distinction is made between normal and abnormal; only the major patient groups are compared among themselves. Therefore, has the Vit D level, which is lower in bipolar patients compared to schizophrenic patients, actually increased? Has it decreased? Or is it normal? The result cannot be deduced here. According to the findings of this study, the result of high Vit D levels in schizophrenic patients can also be deduced, rather than low Vit D levels in bipolar patients. Since no comparison was made with the healthy control group, it cannot give clear information about the relationship between Vit D level and bipolar disorder. The presence of a healthy control group in our study may contribute to the literature in terms of providing information about how Vit D levels actually change in patients with bipolar manic episodes.

Rantala, Luoto, Borráz-León and Krams [7] published a paper where they presented a model that explains ethology of bipolar disorder. It demonstrated that the malfunctioning of the internal clock plays the central role in bipolar disorder causing sleep disturbances. Lack of sleep triggers the mania, by causing an increase in dopamine levels. Stress is often a triggering factor for mania and sleep problems, it also causes neuroinflammation. As inflammation desynchronizes the internal clock, chronic stress and inflammation are the primary biological mechanisms behind bipolar disorder. Since activation of the immune system is known to reduce Vit D levels in the blood, it elegantly explains why authors found mania patients with bipolar disorder have reduced levels of Vit D.

Comprehensive studies on Vit D deficiency in patients with depressive moods have been carried out in the literature, and it has been reported that Vit D deficiency plays an essential role in the pathophysiology of depression [21,22,23]. However, there are still not enough studies on the possible relationship between manic mood and Vit D. A study showed elevated moods were normalized with Vit D supplementation in bipolar manic episode patients [24]. According to limited literature, Vit D deficiency may be associated with manic episodes. A complex mechanism mediates the role of Vit D deficiency in the etiology of mood disorders.

Studies show a relationship between Vit D’s immunomodulatory activity and mood disorders’ neuroinflammatory hypothesis [25]. Vit D exerts an immunomodulatory activity by inhibiting the production of proinflammatory cytokines [12]. Low levels of Vit D may increase proinflammatory cytokines and stimulate the inflammatory response and, therefore, may be an essential factor in the pathophysiology of bipolar disorder.

Gamma amino butyric acid (GABA) is an essential inhibitory transmitter in the central nervous system. A study reported that Vit D deficiency causes an increase in intracellular Ca^2+^ concentration by affecting the GABAergic system, which may have a role in the etiology of bipolar disorder manic episodes [26]. In addition, Vit D deficiency may trigger mood episodes in bipolar disorder through oxidative stress by increasing reactive oxygen species (ROS) [27,28].

Calcitriol, the active form of Vitamin D, activates the gene expression of tyrosine hydroxylase, which is considered a rate-limiting step in the synthesis of neurotransmitters such as dopamine, adrenaline, and noradrenaline [29]. In addition, calcitrol activates the gene expression of tryptophan hydroxylase, the rate-limiting enzyme in serotonin synthesis [30]. These neurotransmitters form the building blocks of the monoaminergic theory, which has an essential place in the pathophysiology of mood disorders [31,32,33,34]. Therefore, Vit D deficiency may play an essential role in the pathophysiology of mood disorders by affecting the synthesis of these critical hormones. A study on young people found no significant difference between the Vit D levels of major depressive disorder, bipolar disorder and a healthy control group. However, this study reported that the number of Vit D-binding receptors is higher in patients with bipolar disorder [35]. In another study conducted with bipolar disorder euthymic period patients, lower Vit D levels were found as compared to the control group [36]. Contrary to this finding, in the current study, no difference was found between the Vit D levels of bipolar disorder euthymic patients and the healthy control group. The fact that we performed Vit D replacement in patients with manic episodes in our study to those with Vit D deficiency may explain this difference. 

The fact that vitamin D levels were lower in all groups in our study compared to Scandinavian countries, which are less exposed to the sun, may be due to less exposure to the sun due to the style of clothing. Therefore, people’s exposure to the sun is more important than the country’s exposure to the sun [37].

In some studies in which the relationship between psychiatric disorders and Vit D were examined, it was documented that Vit D deficiency may not be associated with the pathophysiology of bipolar disorder and that Vit D deficiency may be caused by the deterioration of nutritional patterns and less exposure to sunlight [38]. Consistent with these results, lower Vit D levels in the healthy control group compared to the standard reference values were found due to the probable insufficient geographical exposure to the sun or nutritional irregularities. In future studies, more straightforward information on the relationship between bipolar manic episodes and Vit D may be obtained, by investigating the possible effects of confounding factors such as sun exposure and nutritional status of the patient and control group. 

Based on the idea that comorbid diseases reduce life expectancy and impair psychosocial functioning, various studies have been conducted on comorbid diseases in bipolar disorder [39]. These studies focused on metabolic diseases [40]. There are few studies on the bone health of patients with bipolar disorder [41]. Several studies have reported that bone health is adversely affected in bipolar disorder, independent of comorbid conditions [41,42]. In another study, it was stated that patients with bipolar disorder had low bone mineral densities, this low was correlated with high plasma P levels, and a high P level could indicate low bone mineral density [16]. Contrary to these findings, our findings revealed that there was no difference in Ca and P levels between the bipolar manic episode and the healthy control group. This may indicate normal bone health, even if there is weak evidence. In future studies, hormone levels such as parathormone and calcitonin, which play an essential role in Ca and P metabolism, should be investigated, and bone mineral density should be measured with dual-energy X-ray absorptiometry (DEXA).

## 5. Limitations of the Study

Although we obtained significant positive findings in our study, the results should be interpreted carefully. First, only inpatient manic episodes were included in the study. Second, the sample size was small. Third, essential parameters that may affect Ca, P and Vit D levels, such as the dietary habits and sun exposure time of the patient and control groups were not considered. Fourth, Vit D was added to the treatment of euthymic patients in the study with low Vit D levels, and these patients were included in the study while they were in the euthymic period.

## 6. Conclusions

Our research findings support the evidence that Vit D decreases in the manic episode, which has been reported in a limited number of studies. Decreased Vit D level may trigger a manic episode; insufficient sunlight or malnutrition during the manic episode may cause Vit D deficiency. The evaluation of Vit D, Ca and P levels in the manic episode and euthymic periods of the same patients and the fact that this study is prospective is a crucial aspect that makes the study valuable. Addressing potential confounding factors in future studies and including patient groups with larger samples is essential. 

## Figures and Tables

**Table 1:** Sociodemographic findings of bipolar disorder manic episode patients.

	Bipolar Mania	Healthy Control	*p*-Value ^2^
	*n* = 34 ^1^	*n* = 34 ^1^	
Age	37.82 ± 10.42	35.26 ± 10.79	0.32
Gender			0.81
Female	19.0 (55.9%)	18.0 (52.9%)	
Male	15.0 (44.1%)	16.0 (47.1%)	
Number of manic episodes	4.56 ± 2.51		
Disease duration(year)	11.41 ± 7.86		
Psychotic			
Psychotic	12.0 (35.3%)		
Non-psychotic	22.0 (64.7%)		
Treatment			
Lit + antipsychotic	17.0 (50.0%)		
Valp + antipsychotic	10.0 (29.4%)		
Other mood stabilizers + antipsychotic	5.0 (14.7%)		
Lit + valp + antipsychotic	2.0 (5.9%)		
Vit D supplement	19 (55.9%)		

^1^ Mean ± SD; *n* (%); ^2^ Welch Two Sample *t*-test; Pearson’s Chi-squared test.

**Table 2:** Comparison of pre-treatment bipolar manic episode patients and healthy controls.

	Bipolar Mania	Healthy Control	*p*-Value ^2^
	*n* = 34 ^1^	*n* = 34 ^1^	
Vit D (ng/mL)	10.46 ± 4.93	16.43 ± 5.28	<0.001
Ca (mg/dL)	9.69 ± 0.37	9.70 ± 0.47	0.89
P (mg/dL)	3.31 ± 0.57	3.46 ± 0.46	0.27

^1^ Mean ± SD; *n* (%); ^2^ Welch Two Sample *t*-test; Pearson’s Chi-squared test.

**Table 3:** Comparison of post-treatment bipolar euthymic period and healthy control.

	Bipolar Euthymic	Healthy Control	*p*-Value ^2^
	*n* = 34 ^1^	*n* = 34 ^1^	
Vit D (ng/mL)	15.53 ± 7.90	16.43 ± 5.28	0.58
Ca (mg/dL)	9.79 ± 0.44	9.70 ± 0.47	0.44
P (mg/dL)	3.43 ± 0.62	3.46 ± 0.46	0.86

^1^ Mean ± SD; *n* (%); ^2^ Welch Two Sample *t*-test; Pearson’s Chi-squared test.

**Table 4:** Comparison of Vit D and other parameters before and after bipolar disorder treatment.

	Bipolar Mania	Bipolar Euthymic	*p*-Value ^2^
	*n* = 34 ^1^	*n*= 34 ^1^	
Vit D (ng/mL)	10.46 ± 4.93	15.53 ± 7.90	<0.001
Ca (mg/dL)	9.69 ± 0.37	9.79 ± 0.44	0.19
P (mg/dL)	3.31 ± 0.57	3.43 ± 0.62	0.16

^1^ Mean ± SD; ^2^ Paired *t*-test.

**Table 5:** The relationship between Vit D and sociodemographic variables.

		Age	Number of Manic Episodes	Disease Duration	Vit D
1	Age				
2	Number of manic episodes	0.46 **			
3	Disease duration	0.81 ***	0.72 ***		
4	Vit D	0.08	0.12	−0.05	

Spearman Correlations. ** *p* < 0.01, *** *p* < 0.001.

**Table 6:** Vitamin D, calcium and phosphorus levels according to reference range in groups.

Bipolar Mania		Bipolar Euthymic	Healthy Control	*p*-Value
*n* = 34		*n*= 34	*n* = 34	
Vit D				<0.001
Deficient < 10 ng/mL	19 (55.8%)	10 (29.4%)	0 (0%)	
Insufficient 10–20 ng/mL	14 (41.2%)	14 (41.2%)	26 (76.4%)	
Sufficient > 20 ng/mL	1 (3%)	10 (29.4%)	8 (23.6%)	
Ca				
Low < 8.8 mg/dL	0 (0%)	0 (0%)	0 (0%)	
High > 10.6 mg/dL	0 (0%)	0 (0%)	0 (0%)	
P				
Low < 2.6 mg/dL	2 (5.8%)	2 (5.8%)	2 (5.8%)	
High > 4.5 mg/dL	0 (0%)	0 (0%)	0 (0%)	

## Data Availability

The data used to support the research findings are available upon request from the corresponding author. The data are not publicly available due to ethical restrictions.
